# Digital Twins for Positive Energy Districts: a 10-Step Method for Integrated Design and Optimized Operation

**DOI:** 10.12688/openreseurope.20962.1

**Published:** 2025-09-23

**Authors:** Koldo Urrutia-Azcona, Daniele Salvatore Schiera, Mohammad Mizanur, Giulia Barbano, Niall Byrne, Alexandra Zanasi, Niall Buckley, Benedetta Barchi

**Affiliations:** 1 IES Integrated Environmental Solutions Ltd, Dublin, D01 A8N0, Ireland; 2 Energy Center Lab, Politecnico di Torino, Turin, Piedmont, 10129, Italy; 3 RINA Consulting, RINA, Genova, 16129, Italy

**Keywords:** Digital Twins, Positive Energy Districts, integrated planning, building operation, decarbonization

## Abstract

**Background:**

Positive Energy Districts (PEDs) are central to the European Union’s vision for climate-neutral cities, offering a transformative model for sustainable urban development. However, their systemic, multiscalar, and interdisciplinary nature introduces significant challenges in both planning and operation. Currently, the support of digital twins to PEDs planning and operation is limited due to technological, organizational, and institutional barriers, as highlighted in previous studies. This study explores the role of digital twins in addressing the complexities of PEDs.

**Method and results:**

Accordingly, a step-by-step methodology is proposed to guide the development and implementation of digital twins throughout the PED lifecycle, from early planning to operational management. The methodology emphasizes the integration of technological and spatial dimensions and incorporates user experience research (UXR) to ensure effective stakeholder engagement. The proposed methodology was developed within the context of the PED under development at the Politecnico di Torino campus. This case study demonstrates how digital twins can support system design, simulation, real-time control, and transparent reporting, while also enhancing stakeholder involvement.

**Conclusions:**

All in all, digital twins hold significant potential to overcome the inherent complexities of PEDs, yet their adoption remains limited. The presented methodology offers a structured approach to facilitate their integration, promoting more effective planning, operation, and stakeholder collaboration in PED initiatives.

## 1. Introduction

Positive Energy Districts (PEDs) are emerging as a critical component of sustainable urban transformation, aiming to achieve a net positive local energy balance while fostering broader environmental and socio-economic objectives ( IEA, 2025). Despite growing interest and several attempts ( International Electrotechnical Commission, 2025; JPI Urban Europe / SET Plan Action 3.2, 2020; Wyckmans *et al.*, 2019; Zhang *et al.*, 2021), a universally accepted definition of PEDs has yet to be established, with ongoing debate surrounding, for example, the parameters included in energy balance calculations, integration of qualitative sustainability indicators, and application of corrective factors ( Salom *et al.*, 2021). However, the main conceptual goal of a PED is widely accepted by the scientific community: achieving energy efficiency, sustainability, and community benefits by producing more renewable energy than the district consumes, and integrating seamlessly with the broader energy system ( Gollner *et al.*, 2020).

Currently, some initiatives are exploring the potential of PEDs. Recent findings from the IEA EBC (International Energy Agency’s Energy in Buildings and Communities Programme) within the “ *Sharing the experiences on PEDs: lessons learned from Annex 83*” workshop ( Kozlowska *et al.*, 2024), they underscore the importance of digital twinning technologies as enablers for PED deployment and optimization. Digital twins—virtual replicas of physical systems that allow for continuous data exchange and simulation—support the integrated design, real-time monitoring, adaptive control, and predictive maintenance of energy systems ( Agho *et al.*, 2025). Moreover, the proliferation of digital twins is particularly relevant for managing energy flexibility at the urban scale, where platforms for operational management and strategic planning converge; however, the use of these methods is scarce in PED planning ( Kozlowska *et al.*, 2024). In addition to the technical room for improvement, stakeholder engagement and reporting have been identified as crucial yet often underdeveloped dimensions of PED development ( Kozlowska *et al.*, 2024). A more systematic approach to stakeholder mapping and communication can enhance coordination, transparency and acceptance ( Urrutia-Azcona *et al.*, 2021), ultimately contributing to the long-term success of PED initiatives. 

Despite their substantial potential, digital twinning technologies remain underutilized in the planning and operation of PEDs. While digital twins offer advanced capabilities for system design, simulation, and real-time operational control, their adoption in PED contexts has been limited, often owing to technological, organizational, and institutional barriers. The integration of these tools requires not only technical infrastructure and data interoperability ( Zhang *et al.*, 2021), but also cross-sectoral collaboration and long-term investment strategies that many urban projects still lack. Furthermore, the role of digital twins in supporting stakeholder engagement, a critical factor in the success of PEDs, remains underexplored ( Kozlowska *et al.*, 2024). Beyond their analytical functions, digital twins have the potential to serve as interactive platforms that facilitate communication among diverse stakeholders—planners, policymakers, energy managers, energy providers, and citizens—by visualizing complex data and scenarios in an accessible manner, as well as stakeholders’ reporting possibilities. In this sense, an appropriate user experience (UX) of digital twinning tools is key to fulfilling these shortcomings.

Furthermore, the inherently systemic and multiscalar nature of PEDs necessitates a holistic ad hoc design methodology that addresses technological, spatial, and social complexities at scale from the earliest stages of planning. In this sense, as PEDs operate at the district scale, conventional building-level energy modeling approaches are insufficient. Instead, new frameworks are required to capture the interactions among various energy vectors and networks, which introduce significant challenges in terms of modeling complexity and operational coordination. Considering the aforementioned issues associated with the current application of PEDs at the district scale, this study illustrates the potential of digital twins to alleviate these challenges, which could be beneficial for the proliferation of PEDs.

Accordingly, the aim of this paper is to present a methodology capable of demonstrating how to develop a digital twin at the scale of a PED, a tool that supports both the design and operational phases, while also providing the necessary means for effective stakeholder engagement in decision-making and result reporting processes. To this end, and within the framework of the European Commission-funded Tips4PED project ( Tips4PED consortium, 2025), the study focuses on the case of the PED within the Politecnico di Torino district, which is currently a case study towards energy positiveness.

Regarding the article structure, and following this brief introduction, Section 2 describes the workflow environment in which this methodology is developed, along with parallel research on digital twin user experience. Subsequently, the results section presents the step-by-step digital twinning methodology, showcasing concrete outputs generated using digital twin tools applied to the Torino PED case. Finally, the discussion section reflects on the outcomes and implications of this digital twinning methodology on the PED scale.

## 2. Methods

This study considers the overall approach presented by Marrasso *et al.* (2024), which provides a comprehensive framework for evaluating PEDs in line with the outline of the IEA EBC framework ( IEA, 2025). This approach involves setting specific input-output parameters to guide the analysis. The input parameters included annual data on thermal, cooling, and electric energy demand and supply. The output parameters focus on the energy and environmental (CO _2_) balance from a design perspective as well as the verification of PED goals. The methodology presented in this article extends beyond these initial outputs by incorporating advanced operational tools and parameters to ensure thorough evaluation and achievement of objectives throughout the lifespan of the PED.

### Process for a PED digital twinning method; from design to operational stages

The main objective of this article is to present a method that establishes a holistic approach to the design and operational stages of a PED, from the initial selection of a district and the definition of its physical boundaries to a stage where district managers can implement and monitor interventions as well as assess their actual or potential impact on the district’s energy positiveness, thus setting an ongoing and iterative cycle towards achieving the ultimate goal. On the one hand, by “design” stage, the authors refer to all tasks that aim to create a robust and validated digital twin PED baseline, where physics-based simulations can be launched upon that baseline to assess which set of interventions would be the best towards the district’s energy positiveness. On the other hand, the “operational” stage refers to the activities focused on providing reliable data display and analysis for ongoing energy management decisions across the lifespan of the PED.

Regarding the design stage specifically, the method presented in this article significantly overlaps with Barbano et al study developed for the Brussels airport case ( Barbano *et al.*, 2024), which can be considered as a very specific case of PED. For both pieces of research, the projects in which the methods were developed and applied followed a performance measurement and verification (PM&V) methodology for decarbonization, starting with establishing a baseline through the evaluation of sustainability metrics. This design process involves data collection for buildings and district networks, both static and dynamic, including energy consumption and CO _2_ emissions, which are used to create a detailed digital twin model that is later validated against historical data to ensure accuracy. This model supports the evaluation and planning of decarbonization projects, ensuring a robust and reliable baseline. Specific actions and interventions were planned and deployed with ongoing measurements to assess performance. Furthermore, and more relevant to this article, the method sets a digital twinning approach that supports this by creating virtual replicas for scenario evaluation and decision making, leveraging physics-based building simulation models coupled with real data, and setting the crucial elements of how a digital twin can support PED design.

In terms of the operational management of PEDs, the methodology strategically leverages the capabilities of cloud-based simulation engines to improve the operation of buildings based on insights provided by day-ahead recurrent simulations. These simulations provide critical insights into potential system behaviors and performance under various scenarios, allowing for proactive adjustments and the optimization of operational services. As a result, the integration of cloud-simulation technologies not only improves forecasting accuracy but also supports more resilient and adaptive operational management.

### Tools for building a PED digital twin

Currently, the use of digital twinning tools to design and operate a PED is highly recommended because of its ability to recreate the main elements of a real counterpart, digitally encompassing complex realities and processes, and delving into a level of detail beyond the reach of more traditional planning approaches. In this sense, digital representation, simulation, and real-time management play a critical role in supporting PED transition by enabling data-driven decision-making, optimizing energy flows, and enhancing the ability to plan, monitor, and adapt interventions dynamically across districts.

Accordingly, this study integrates such types of tools within the methodology presented in the Results section, leveraging the IES digital twin suite of tools demonstrated in greater detail in previous studies ( Barbano *et al.*, 2024; Buckley *et al.*, 2024; Byrne *et al.*, 2024). For the design stage, this research uses intelligent Community Design (iCD), intelligent Virtual Network (iVN), the iSCAN cloud platform for data management and analysis, and the cloud intelligent Community Information Model (iCIM). These tools collectively support the creation, validation, and optimization of community-scale digital twins, enabling comprehensive energy, carbon, and economic building analysis at the PED level. Furthermore, to reinforce the building energy operation and management stage within the Torino PED, this study employed two additional tools. First, the Virtual Environment (VE) is utilized for an in-depth integrated analysis of energy design and retrofitting at the building level, leveraging the sharpest building physics-based simulations possible through the Apache engine (extensively tested and used in research studies such as Ahmad *et al.*, 2017; Chang *et al.*, 2024; Mane *et al.*, 2020; Reeves *et al.*, 2015). In the case of the Torino PED, VE is used to perform a detailed analysis of those buildings that are meant to be managed in real time by a building portfolio energy manager, intending to ensure the highest possible accuracy level. Second, IES Live software was introduced to optimize the operational performance of each of those buildings throughout their entire lifecycle, allowing operation at a building portfolio scale for the energy manager via cloud-based dashboards. The IES Live data-driven approach is key for the operation of Torino PED as it identifies the most impactful building management and upgrades, maximizing energy efficiency, carbon reduction, and both capital and operational savings.

**Figure 1.**
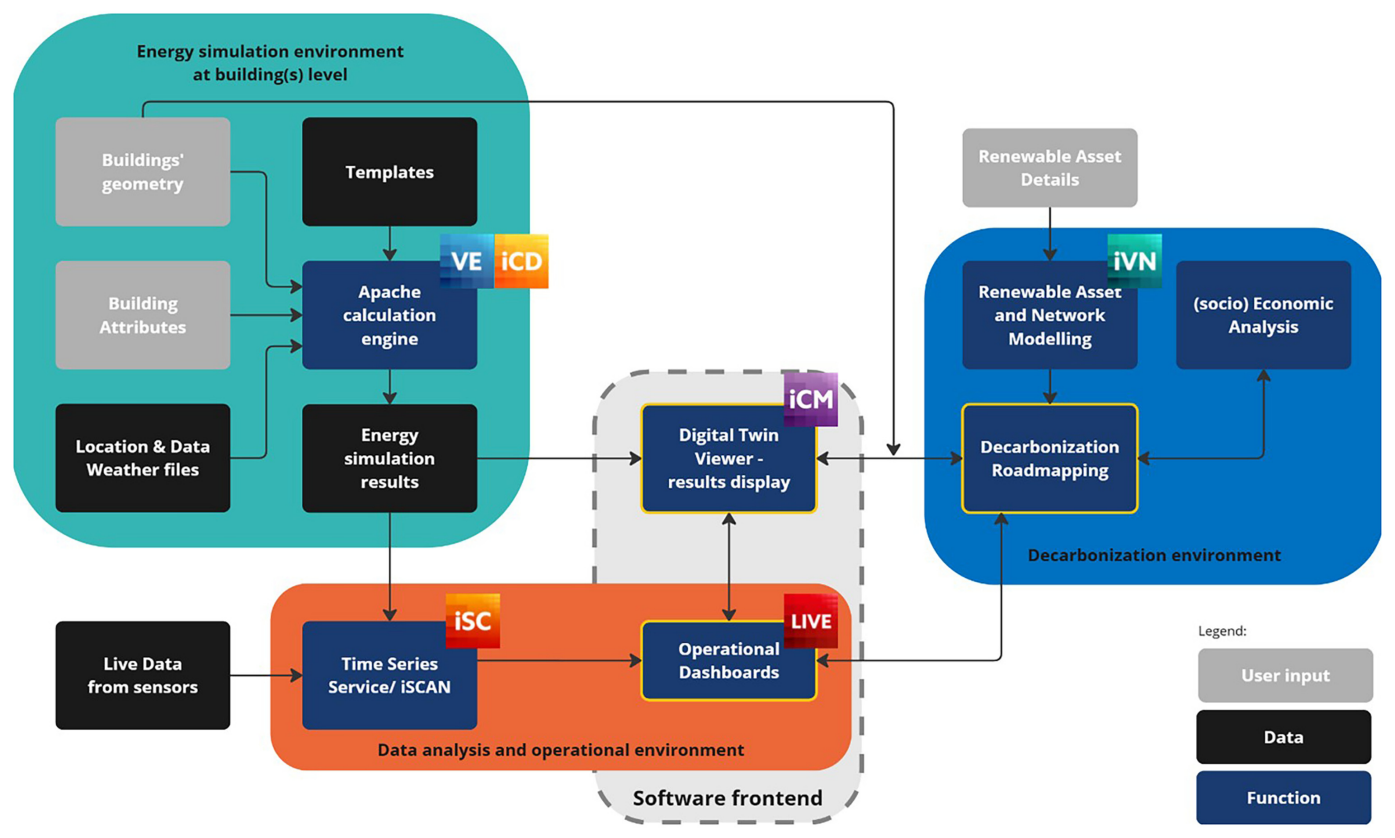
Workflow supporting the PED digital twinning methodology.

The use of each of these tools is further detailed in the results section, along with its specific stage and context within the digital twinning PED methodology. To provide a first overview, Figure 1 shows how all these tools interact with each other within the overall digital twin workflow.

### User experience (UX) research guiding digital twinning tools

When designing tools for constructing a digital twin of a PED, it is crucial to prioritize the user experience throughout the process. To achieve this, a comprehensive approach was adopted, encompassing the development of user experience to consider the specific needs of different user categories. User experience research (UXR) is a part of product development focused on understanding what users need from a digital product, as is the case with digital twinning technology, and how they would use it in real-world contexts. A common misconception is that UX work begins with designing a user interface (UI); however, understanding user needs early helps shape the project scope, prioritizes high-impact features, and guides infrastructure or technology choices. 

A major strength of UXR is its ability to reduce the risk of cognitive bias in software development, which can often reduce the intuitiveness or functionality of the software for the end user. This lack of UXR can distort the software development team’s understanding of users’ interactions with systems, leading to lower levels of users’ adoption, learnability issues, potential rework situations, and loss of trust in the software product.

This is why this research introduced UXR in the early stages of development in two main phases. First, within *generative research*, the focus was on understanding the target audience and identifying opportunities, delving into *who we’re designing for* and *what problem we’re solving*. Second, through *evaluative research*, tests and prototypes checked if the software solutions were on the right track, focusing on *how* the solutions performed in detail. Within the generative research stage, the team conducted semi-structured interviews in a conversational tone with the targeted personas as potential users of the software product and digital twinning methodology:

- The energy manager of a university campus – Politecnico di Torino
- The energy manager of municipal buildings – City of Turin

These two stakeholders were chosen as they manage energy assets that can be assimilated as a PED case, such as a university campus containing multiple buildings and energy networks and the building portfolio of a municipality, which also shares common elements with the conceptualization of a PED. Furthermore, a questionnaire focusing on the jobs-to-be-done by these types of energy managers was created to understand their daily duties and challenges, clustering their answers to obtain software development insights. This questionnaire was completed by six energy managers across Europe (i.e., universities, municipalities, portfolio energy managers), and together with the interview insights and a workshop celebrated with Turin PED stakeholders, they were all key to guiding the product software solutions developed to back up this digital twinning methodology.

As a result of this UXR, the process identified insights into user preferences, usage patterns, and pain points, helping designers to identify the features that are most important to include in the tool, as well as organizing workshops and creating mockups as visual representations of the tool to illustrate its design and functionality. These serve as prototypes that can be tested by users to gather feedback. This iterative process allows for adjustments based on user input before final product development. This user-centered approach enhances the effectiveness of energy planning and operational strategies within the district, ensuring that they are not only technically sound but also aligned with the needs and expectations of the community. The activities performed were tailored specifically for the unique context of the Turin PED analysis, considering the diverse and distinct requirements of each stakeholder group within the district. This includes municipalities that are responsible for governance and infrastructure, energy managers who oversee energy efficiency and sustainability initiatives, and residents who are the end-users of energy systems. By engaging with these stakeholders through targeted research and interactive sessions, the research team gathered valuable insights into the design and functionality of the digital twin.

### Turin PED case study

To effectively demonstrate the digital twinning methodology, a specific urban district in Turin was selected as a representative case study. This selection highlights the unique characteristics and challenges of the area and serves as a practical example to showcase the application of the methodology and technologies exploited in a real-world urban context. Moreover, Turin has been recognized as one of the 100 cities participating in the European Commission’s ambitious NetZeroCities Mission ( European Commission, 2022), a groundbreaking initiative aimed at accelerating the transition to carbon neutrality across urban environments. Within this framework, Turin has designated a specific PED area that serves as a lighthouse site under the European project Tips4PED ( Tips4PED consortium, 2025). This project seeks to explore and implement innovative strategies to achieve energy efficiency and sustainability in urban settings. By focusing on this district, we can examine the complexities of urban dynamics, infrastructure performance, and potential of digital twinning to improve urban planning and management strategies.

**Figure 2.**
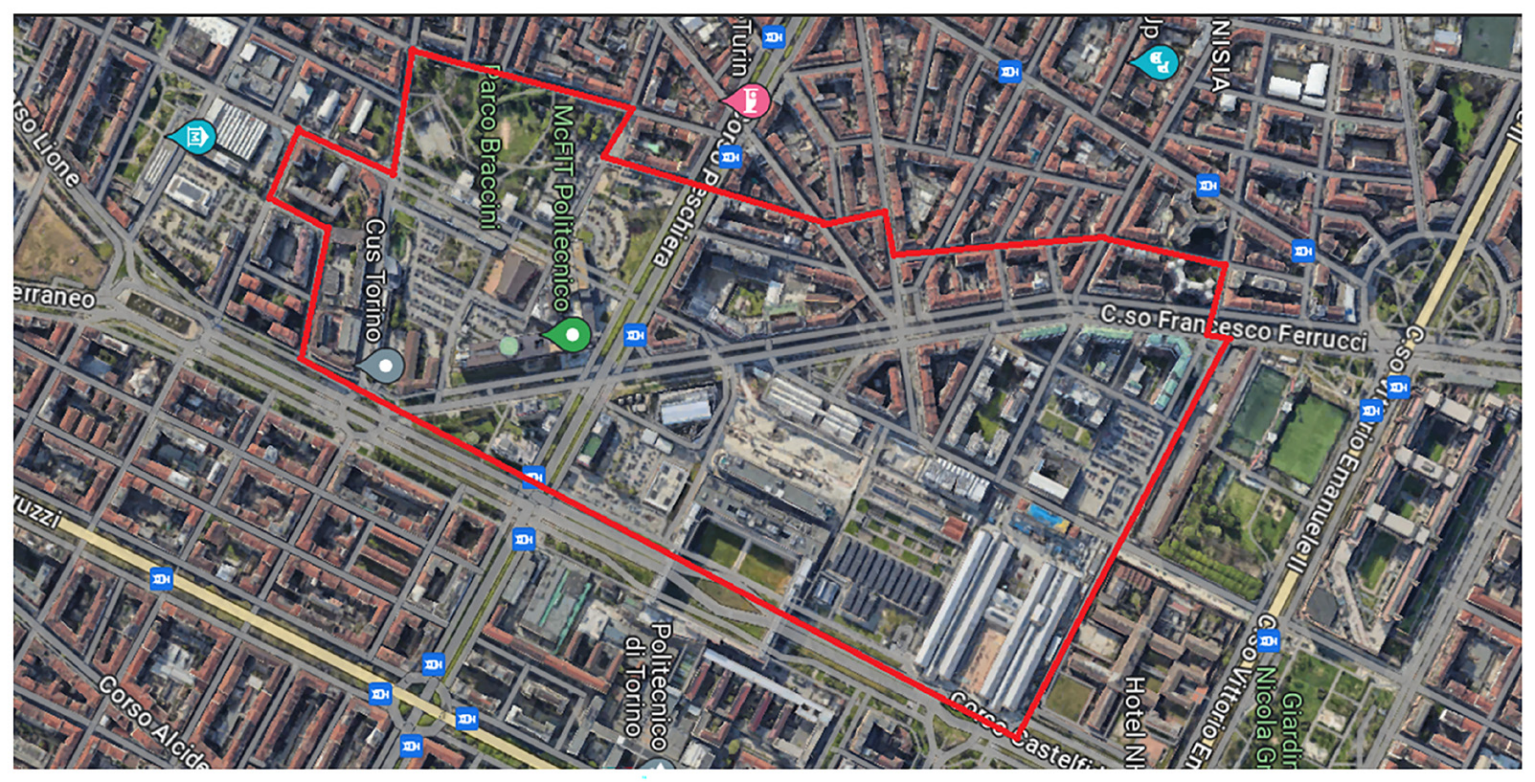
Turin PED boundary (Google Earth caption).

The Turin PED encompasses an area of approximately 260,000 square meters ( Figure 2), integrating the main campus of the Politecnico di Torino. This location serves as a vital urban hub, characterized by a rich tapestry of educational institutions, cutting-edge research facilities, municipal services, and residential buildings. In addition to electrical and gas networks, the PED also features a district heating network, enhancing its infrastructure and sustainability. The diverse nature of this site makes it an exemplary candidate for implementing a PED, as it showcases a heterogeneous building stock, multifaceted energy infrastructure, and a variety of mixed-use actors and stakeholders. Within the boundaries of the PED, several key components contribute to its functionality and energy portfolio.

- Educational and Research Buildings: The district is home to a range of educational facilities, including classrooms and laboratories, that foster innovation and learning. Notably, the Energy Center building serves as a focal point for energy research and development, while Cittadella Politecnica enhances the collaborative environment between students and researchers.
- Municipal Infrastructure: The PED incorporates essential municipal services, such as the municipality’s sports and employment offices. These facilities support local governance and promote community engagement in the districts.
- Residential Assets: The district includes three university dormitories providing accommodation for students, as well as nearby private residential buildings. This mix of housing options ensures a vibrant community atmosphere, fostering interactions between residents and students alike.
- Energy Systems and Infrastructures: PED energy needs are covered by diverse energy systems and infrastructures. The building stock is equipped with diverse energy technologies for space heating and cooling and domestic hot water, such as gas boilers, electrical boilers, and heat pumps. Moreover, integration with Turin’s district heating network enhanced the diverse heating solutions available in the area. In addition, the PED area includes rooftop photovoltaic systems, which collectively generate a substantial 1.7 MW of renewable energy across the district. In addition, the presence of groundwater heat pumps further diversifies the energy portfolio.

## 3. Results

**Figure 3.**
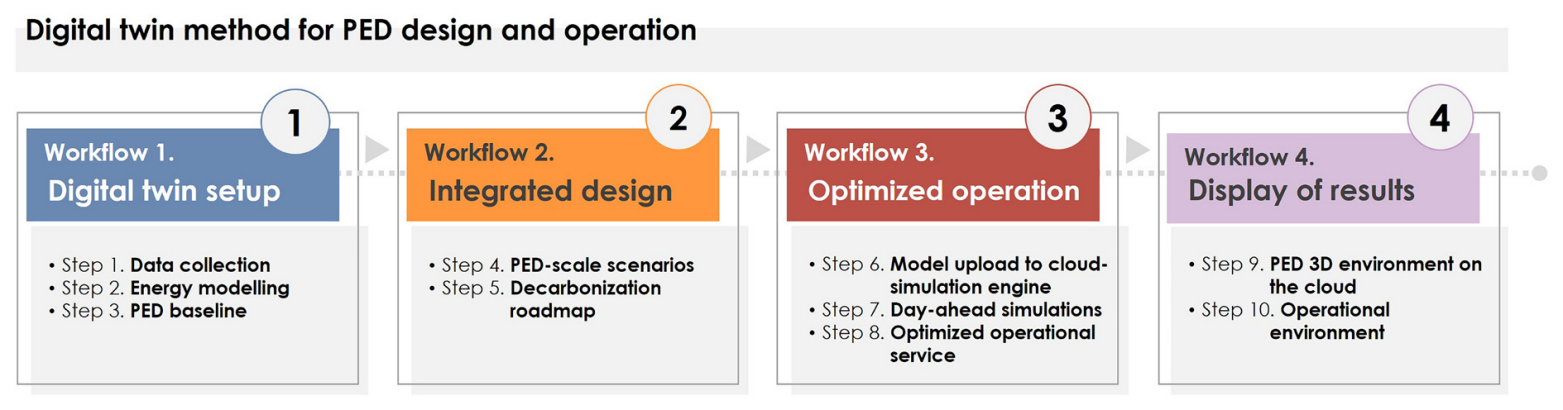
Digital twinning methodology structure.

This section presents a step-by-step digital twinning method developed to facilitate the integrated design and optimized operation of PEDs. The methodology is structured in four different stages ( Figure 3), intending to draft a comprehensive user journey from data collection to the display of results:

- *Workflow 1* focuses on setting the digital twin up, including data collection, modeling, and validation tasks, achieving a robust baseline of the PED for the design and operational stages.
- *Workflow 2* addresses the integrated design of PEDs through the simulation of Energy Conservation Measures (ECM) packages, which support the creation of a PED decarbonization roadmap.
- *Workflow 3* delves into the optimal operation of buildings and networks as well as running automatic predictive performance simulations to facilitate maintenance and operational recommendations.
- *Workflow 4* presents the display of PED’s digital twinning results based on a 3D-inmersive environment and operational dashboards, both rooted on easily accessible on-the-cloud platforms

## Workflow 1 – Digital Twin setup

### 1. Data collection

The digital twin setup starts with the data collection process, which entails two main sources of information. First, a set of static data, which does not necessarily vary over time, provides the key PED characteristics for modeling purposes, such as building location, geometry (i.e., height, footprint), thermophysical properties (i.e., energy performance certificates [EPC]), and equipment installed (i.e., HVAC systems). Second, dynamic data in the form of time-series metrics retrieved by IoT sensors installed on specific buildings, including measurements such as live energy consumption, CO _2_, temperature, occupancy, and other key parameters, are used to understand how the buildings perform.

In the case of Turin, static data were collected within a single geopackage file at the PED scale, integrating diverse sources of information in Geographical Information Systems (GIS) format. In particular, the territorial GIS database was exploited to retrieve the PED building stocks, footprints, typologies, and uses, while the thermophysical characterization of the buildings, energy performance, primary energy consumption, and supply technology data were obtained from the analysis of the EPC and Energy System Register databases. Furthermore, for specific buildings intended to be monitored and subject to operational services, more detailed information such as Building Information Modeling (BIM) files is required. Regarding dynamic data, both the existing sensors already displayed within the PED area and the new sensors to be installed according to the PED project objectives must be channelled through a dynamic data architecture that structures the ontology, data collection protocols, sensor decoding, data storage, and appropriate communications to enable the operational services of the digital twin.

Finally, a crucial element of the data collection process is weather information. For this purpose, the digital twin requires weather files (that is, EnergyPlus Weather -.epw- files) to ensure that outdoor environmental conditions mirror the real environmental conditions of the physical district as accurately as possible, hence providing the required environment to calculate heating and cooling demands. In the Turin case, the weather files were obtained from the open-source repository climate.onebuilding.org ( Climate.OneBuilding, 2025) for the baseline, which can be later adjusted to create future weather files for future simulated scenarios within the PED area.

### 2. Energy modeling at PED scale

**Figure 4.**
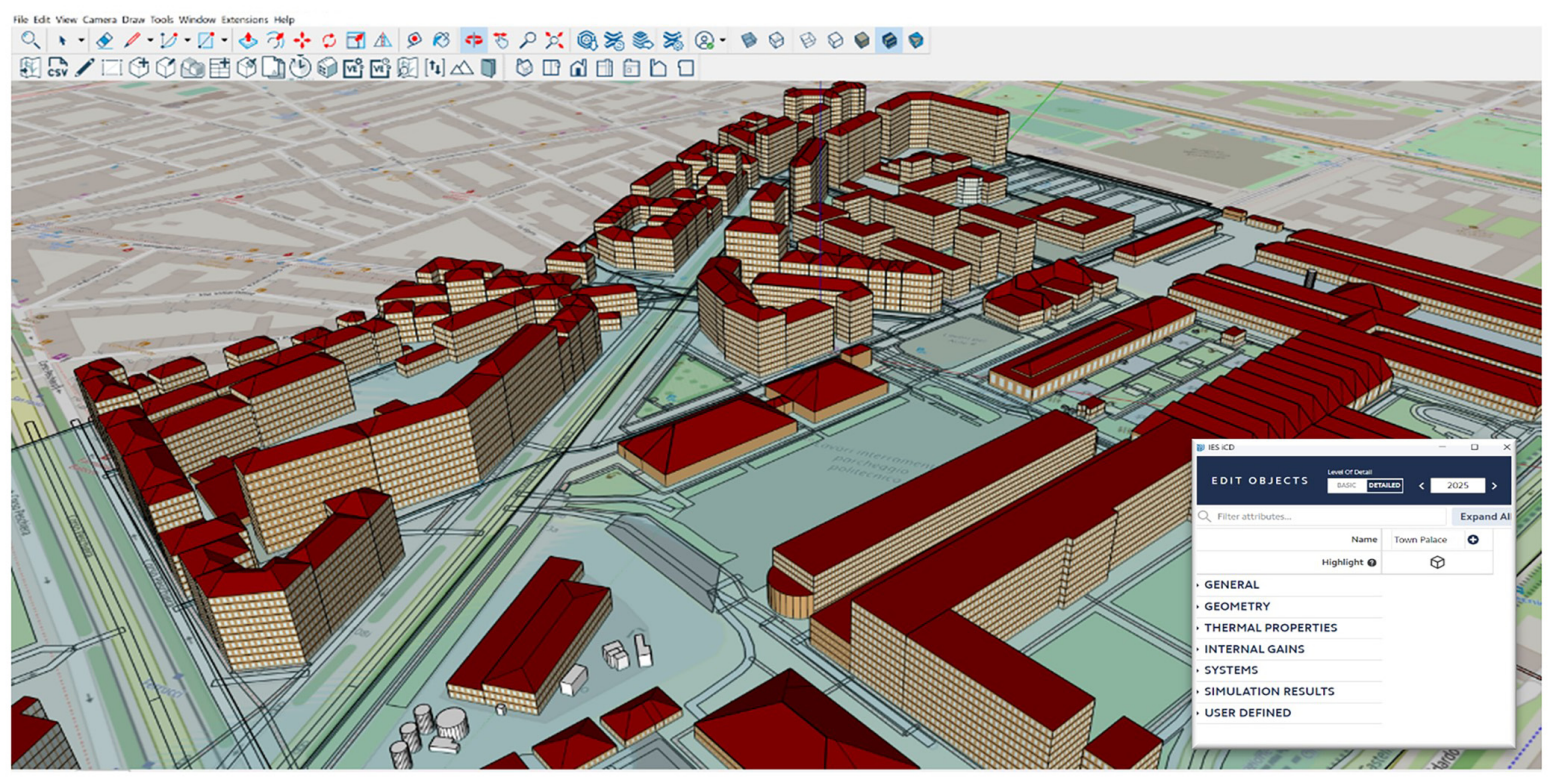
Modeling of the Turin PED in iCD software.

Once a comprehensive set of data has been collected, it is processed as an input for the iCD software, adjusting the building parameters to design the physics-based model at the PED scale ( Figure 4). For model creation, the geopackage data collected are synchronized and sharply appointed to every single building in the area, with specific values for diverse attributes, such as primary energy use, construction date, number of stories, height, thermal properties, internal gains, and equipment (i.e., HVAC, lighting), which are critical for energy simulations in later stages.

**Figure 5.**
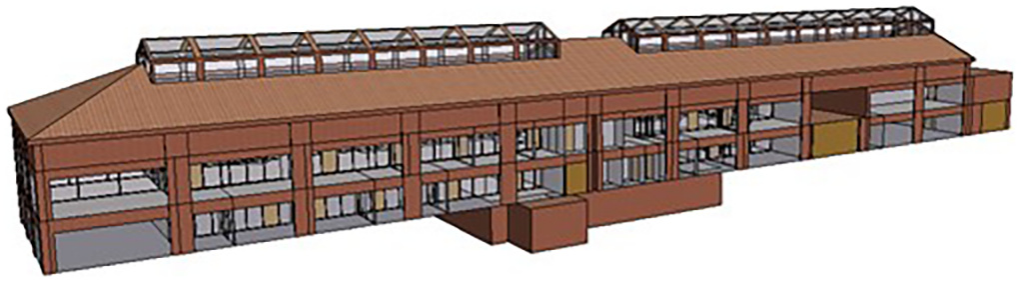
Modeling of the Braccini building, part of Turin PED, in VE software.

Furthermore, to enable a more detailed analysis and operation of key buildings within the PED, VE software was used to thoroughly model the buildings leading to operational services (i.e., Figure 5). In this case, the modeling criteria are much more granular than those in the iCD, hence requiring extensive input data to accurately represent the digital replica of the building. For this process, the building geometry is based on architectural plans, including critical information such as building thermal fabric characteristics, occupancy profiles, internal gains, and HVAC systems for each space. Within the Turin PED, some buildings have been identified as suitable assets for operational energy services. Accordingly, BIM files of these buildings were collected and exported through a semi-automated process to the VE software, using the Pollination Revit plugin for this transfer of information ( Pollination, 2025). Regarding the sequence of this digital twinning methodology, it is convenient to stress that the iCD modeling will feed *Workflow 2 – Integrated design at PED scale*, while the VE modeling will feed *Workflow 3 – Optimized operation at building and building portfolio scales*.

**Figure 6.**
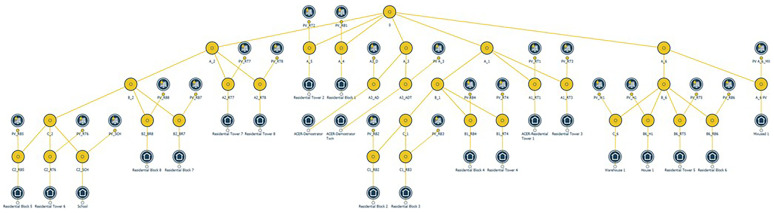
Example of electrical grid modeling in iVN software.

Within the PED context, not only buildings but also energy networks are crucial elements for energy positivity. In this case, the energy data from each building were imported into the iVN software to evaluate district network loads and understand the baseline grid demand ( Figure 6). Based on this background, the PED manager will be able to test grid connections over different scenarios to understand the increase or decrease in grid demand and how to cope with such changes. For example, and as a general statement to be covered by such analysis, the more EVs penetrate the mobility share and renewables are connected to the PED, the more exhaustive the network analysis must be to meet such electrification parameters through iVN.

### 3. PED baseline simulation & validation

With the PED model already built, the next step focuses on achieving the most accurate baseline model at the building and PED scales, setting a solid background for future energy simulations and scenarios within *Workflow 2 - Integrated design at PED scale*. If building energy consumption metrics are available, they can be used to calibrate the model to ensure that each digital building replica performs according to its real counterpart ( Tardioli *et al.*, 2020). At the PED level (modelled in iCD software), buildings can be validated against their real EPC values, whereas at the building level (modelled in VE software), each can be validated using monthly energy data according to the ASHRAE Guideline 14 ( ASHRAE, 2014). In this regard, the equation below and Table 1 show the allowable tolerances between virtual models and real consumption data, where monthly calibration needs to achieve ±5% mean bias error (MBE) and a coefficient of variation of root mean square error Cv(RMSE) of 15% (a good practice for validated buildings in the iCD model is to achieve ±10% energy variation). After this process, the model is ready to accommodate future scenarios based on the validated baseline.

**Table 1.** ASHRAE Guideline 14 Acceptable calibration tolerancse ( ASHRAE, 2014).

Calibration type	Index	Acceptable Value
Monthly	MBEmonth CV(RMSEmonth)	±5% 15%
Hourly	MBEmonth CV(RMSEmonth)	±10% 30%

Equation for measured energy consumption:

$$\text{MBE}\left(\%\right)=\frac{{\text{Period}}^{\sum {\text{(S-M)}}_{\text{Interval}}}}{\sum_{\text{period}}{\text{M}}_{\text{interval}}}\times 100$$

Where M is the measured kilowatt-hours or fuel consumption during the time interval; and S is the simulated kilowatt-hours or fuel consumption during the same time interval.

## Workflow 2 – Integrated design at PED scale

### 4. PED-scale scenarios

Once the digital twin has been validated for accuracy against measured data, it can be considered to be fit for purpose, and the next workflow in the process can take place. The workflow was divided into two steps. The first step, described in this section, covers the definition and analysis of suitable Energy Conservation Measures (ECMs) that can be implemented in a district towards achieve PED status.

This stage begins with the definition of ECMs suitable for the district, its buildings, and infrastructure. This requires an initial expert scoping of technologies and solutions available in the local market, and is appropriate for the architectural and environmental context of the district. In some contexts, there may be existing Climate Action Plans (CAPs) at national or local levels, which set out both emission reduction targets and mitigation and adaptation strategies to adopt, which should act as a basis for the analysis carried out with the digital twin ( European Commission, 2021; Palermo *et al.*, 2020).

The ECMs identified can range from shallow interventions, which require minimal discomfort to building users and relatively small investments (i.e., substituting obsolete lighting with LED bulbs), to more intrusive but relatively quick deeper interventions (i.e., replacing windows for higher insulation, installing solar systems both for electricity and thermal energy generation), up to full renovation interventions that require longer work, higher investment, and potential user displacement (i.e., nearly zero energy building nZEB-level insulation, HVAC system replacement) ( Semprini *et al.*, 2017). The solutions for several climates and contexts are well known in the literature, and their identification is beyond the scope of this study ( Byrne *et al.*, 2024).

Once a list of candidate ECMs has been identified for the PED, they should be organized into groups that collect the application of similar actions across several buildings in the district and the overall implementation timeframe. These actions could also include interventions at the PED level (i.e., the installation of distributed energy generation and storage). At this stage, if possible, the cost of the interventions should also be estimated from the literature or past projects in the district to enable cost/benefit analyses in later stages. Where relevant, any requirements on the order of application of the ECMs should be identified. For example, the replacement of high-temperature heating distribution systems should occur before the installation of low-temperature heating generation systems (i.e., heat pumps).

**Figure 7.**
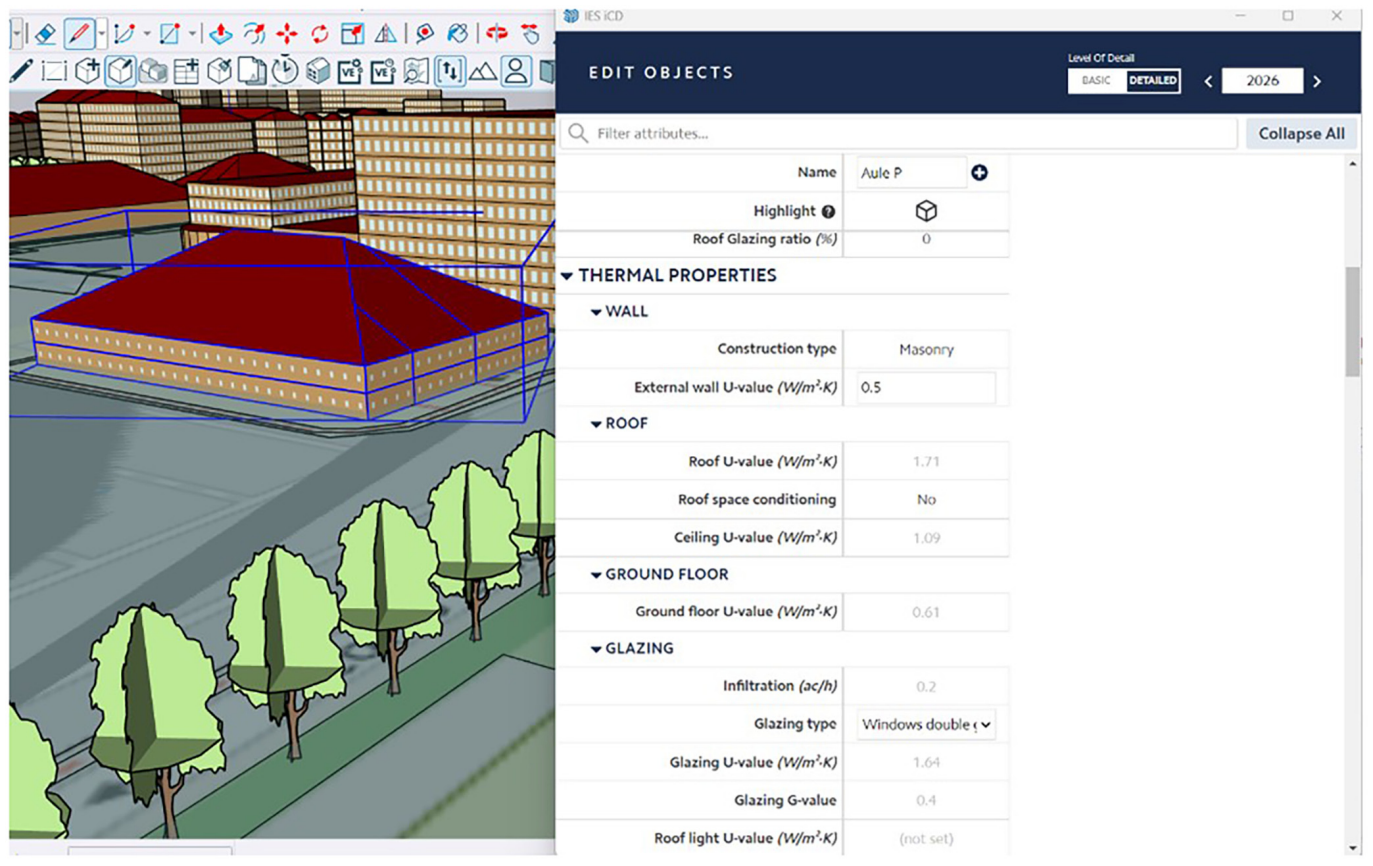
Configuration of ECM in iCD software for baseline and future scenarios.

The identified ECMs actions can now be analyzed for their impact across the implementation timeline using the calibrated digital twin created in the previous workflow stage. In practical terms, this means modeling the ECM on top of the baseline digital twin for the implementation year ( Figure 7). Depending on the type of ECM and the level of detail of the existing digital twin, this analysis can be performed using either VE or iCD software. When there is a strict dependency on the order of execution of the ECMs, this should be considered in the modeling.

Once the simulations for each of the ECM actions were carried out for each building or group of buildings, the simulation outputs by year and ECM must be collected and divided by energy consumption, CO2 emissions, and energy costs. These outputs, in the form of time series per ECM action and area, can be aggregated into yearly values to be collated into decarbonization pathways for decision-making support.

### 5. Decarbonization roadmap

Once the impact analysis results are available from the digital twin, the second and final steps of the workflow can be performed. This step, described in the present section, focuses on the creation of a decarbonization roadmap for decision support and future tracking.

The savings estimated by the digital twin for each intervention can be mapped on a timeline to compare the evolution of the PED’s potential performance against the baseline defined in the previous workflow, which acts as the Business as Usual (BaU) scenario. At this stage, the interventions can be grouped into different scenarios to represent the varying levels of investment capability in the PED, legal constraints and requirements, and funding opportunities. This analysis requires the input of the PED manager, who is best placed to identify these boundaries and to define the nature of each scenario.

**Figure 8.**
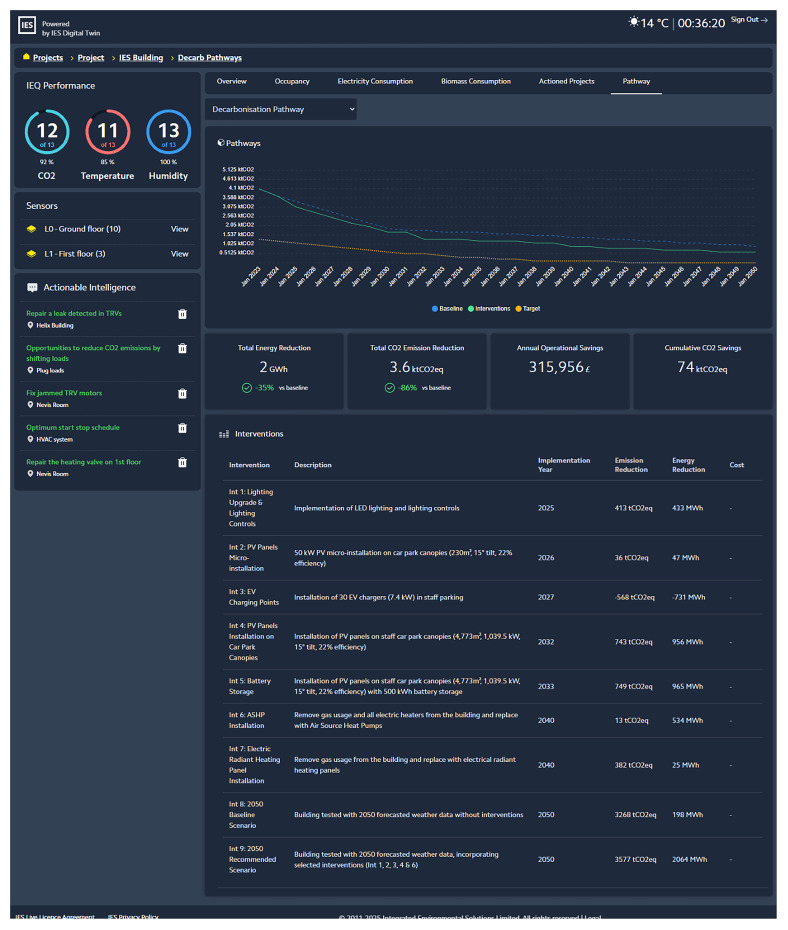
Decarbonization roadmap for 2025-2050 in IES Live software.

The scenarios can then be compared in IES Live to explore their potential performance pathways on several metrics of the environmental and economic impacts as shown in Figure 8. This comparison offers decision support to the PED manager, who can decide together with other relevant stakeholders on the most suitable decarbonization pathway to pursue in the district.

The final scenario selected becomes the decarbonization roadmap for the PED and remains available for ongoing monitoring and tracking, as the ECMs are deployed on the PED. The actual ongoing performance of the PED should be tracked for comparison with the milestones in the roadmap to enable the PED manager to detect deviations and promptly reevaluate new scenarios as needed.

## Workflow 3 – Optimized operation at building and building portfolio scales

### 6. Model upload to cloud-simulation engine and configuration

The requirement for ongoing optimized operation at the building and portfolio scales requires the use of a cloud-based physics engine (Apache-on-the-Cloud – AoC) as a means of implementing recurring simulations of the digital twin assets. At the building scale, having been identified and modelled in Workflow 1 using the VE software package, key demonstration buildings, ideally comprising one building per archetype, are modelled locally using the VE, and an Apache input file (APR file) is created from the desktop version of the software. Once created, this APR file is ready to be consumed by the AoC engine, and it is uploaded to the AoC platform, where it is available for simulation and optimization when triggered.

These individual building models are then federated on a single digital twin platform to represent the portfolio of buildings on a nodal-network platform that leverages the iVN software. This tool enables the user to examine the resource demand requirements for each of the pilot buildings on a single pane-of-glass and examine network constraints based on the forecasted demand profiles. The building-level digital twin is linked to a corresponding building within the iVN network model and then connected to its associated resource node (electricity substation, district heating generator, etc.) along with other buildings connected to the targeted node. The forecasted demand profiles for each building were then aggregated to indicate the demand at the node level, enabling the user to undertake optimization at the building level and examine the impact on the district node, ensuring that demand can be appropriately satisfied on the supply side.

### 7. Day-ahead recurrent simulations and Time Series Service

Once the chosen archetype buildings are uploaded to the AoC platform, the user can initiate a recurring simulation protocol for each of the chosen buildings through a Graphical User Interface (GUI). In the case of this workflow, this will involve the specification of daily simulations that occur at a fixed time (generally 18:00h) each day and will leverage the calibrated building digital twin model in the cloud-based physics engine to forecast the demand profile for the following day using forecasted weather profiles generated through a dedicated weather service. A separate optimization algorithm is also provided within the platform that enables the user to optimize the demand profile at either the building or network level based on a predefined constraint. For example, if the user wishes to minimize their energy costs, this can be represented as the most important constraint, and the objective function will apply appropriate weighting to this constraint during its computation. The optimization algorithm leverages the simulated demand profiles for the buildings and adjusts them based on the constraints to satisfy predefined user requirements for the building or network.

The data layer on which the digital twin platform sits is called the Time Series Service (TSS), and is accessed through a dedicated API that links directly to the AoC platform. When the APR files for each building digital twin are first uploaded to AoC, the TSS automatically generates a data channel for that specific building. The user can then interact with the TSS channels to specify the post-processed simulation data that are of most interest to them and their specific use case. For example, if a specific data point or KPI (i.e., Energy Use Intensity) is not automatically generated through the simulation, the user can define its own specific KPI based on its calculation process through an “Expression definition” wizard. This will then create an additional data channel for the building or buildings of interest that will automatically calculate this KPI, as new data are generated through simulation. In addition to defining custom KPIs, the user can specify the time steps in which each data channel should report the values for. This can vary from 6-minute to 1-hour intervals based on the nature of the data channel and the use-case requirements of the user. This process is completed for each building uploaded to the AoC service. Once the recurring simulation is triggered, as outlined previously, the data output from the simulation is transferred automatically via the API to a specific data channel for the target building within the TSS platform.

### 8. Optimized operational service

**Figure 9.**
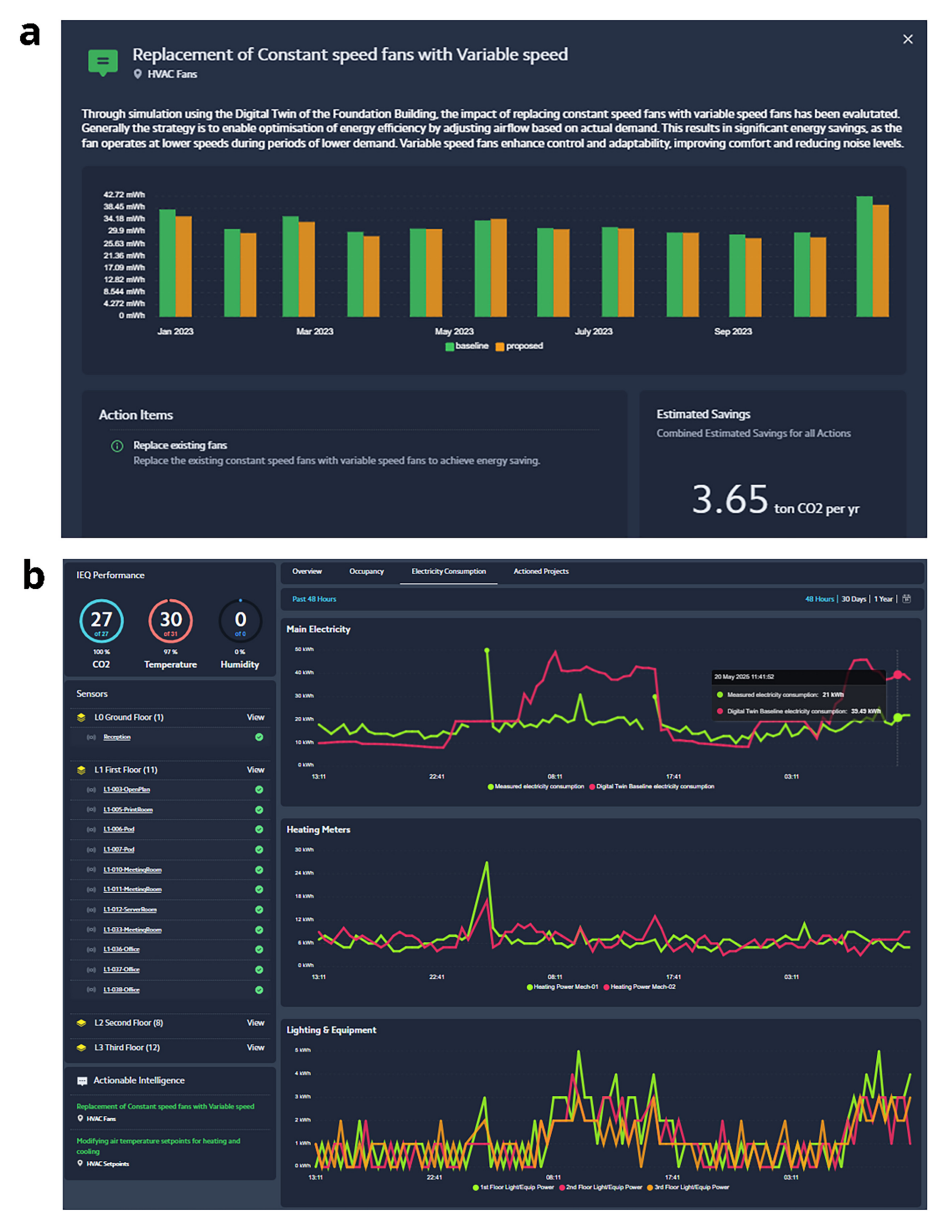
Actionable Intelligence and overview of electricity consumption within IES Live dashboards.

Having set up the requirements for recurring simulations in AoC, the initiation of the simulation process and associated data optimization and transfer is a fully automated process that is triggered at a predefined time daily. The simulation run was initiated at a specific time. AoC automatically accesses the external weather service to access the forecasted weather file the day ahead. Thereafter, the APR file from the previous day is accessed, and the simulation begins, with the demand profile for the following day provided as output from the simulation, including HVAC setpoints, stochastic appliance operational profiles, and other demand-related information that will contribute to the building’s resource consumption the following day. This process was undertaken for each of the buildings that had APR files uploaded to the AoC platform. Each of these data channels is transferred to the TSS via the API scripts and made available for optimization by a dedicated demand optimization algorithm. This algorithm is triggered either at a prespecified time or upon the transfer of demand data to the TSS. The user-defined constraints for each building, as well as for the network itself, are used within the algorithm’s objective function to adjust the building-level demand profiles based on the required output of the process, for example, to maximize the self-consumption of renewables, minimize operational costs, or maximize internal thermal comfort. Once the optimization process is terminated, the optimized demand profiles are transferred to a dedicated data channel within the TSS, which is then accessible by the iVN network model. This optimized network model then presents the required resource supply to satisfy both the baseline and optimized demand profiles of the buildings. Finally, the resulting insights, can be displayed in IES Live dashboards for operational purposes ( Figure 9).

## Workflow 4 - Display of results

The previous workflows of this methodology are focused on processes, setting the basics of the digital twin to later develop design and operational tasks in the PED area; now it is time to display the results of such workflows. The chosen formats for both design and operational result display are on-the-cloud platforms, which remain accessible through a simple URL, but with the possibility of assigning tiered access to information and editing permits depending on the user (i.e., PED manager vs. citizen).

### 9. PED 3D environment – iCIM on-the-cloud platform

**Figure 10.**
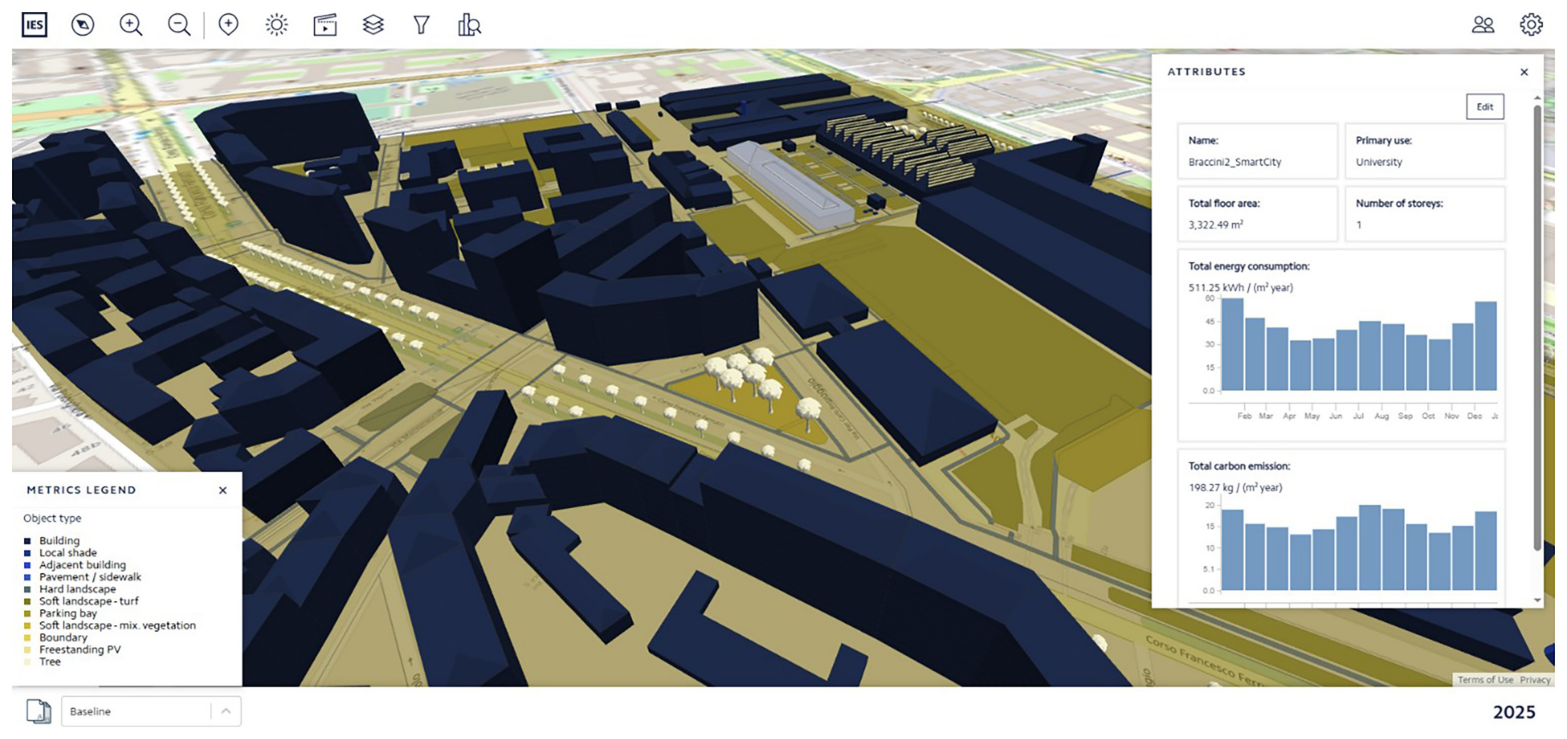
Digital twin 3D-immersive environment displaying Turin PED results within iCIM on-the-cloud platform.

The outcomes obtained from Steps 3 ( *PED baseline simulation & validation*) and 4 ( *PED-scale scenarios*), both developed with the iCD desktop-based tool, can be synchronized and finally displayed on the iCIM cloud-based platform, making it easily accessible to any user. This platform shows a 3D-immersive environment with all building information and static data simulated for both the baseline and future scenarios, even displaying graphical and metric differences among them. Figure 10 shows an example of such a 3D environment displaying the Turin PED results.

Regarding this 3D-immersive environment, it is relevant to stress the possibility of embedding URLs into the attributes of each building displayed on the platform. This possibility enables a seamless connection between the 3D environment and the dashboards per building, or a portfolio of buildings, as described in the next step ( *Operational environment*), creating a smooth transition from design to operational services from a UX point of view.

### 10. Operational environment – IES Live on-the-cloud platform

**Figure 11.**
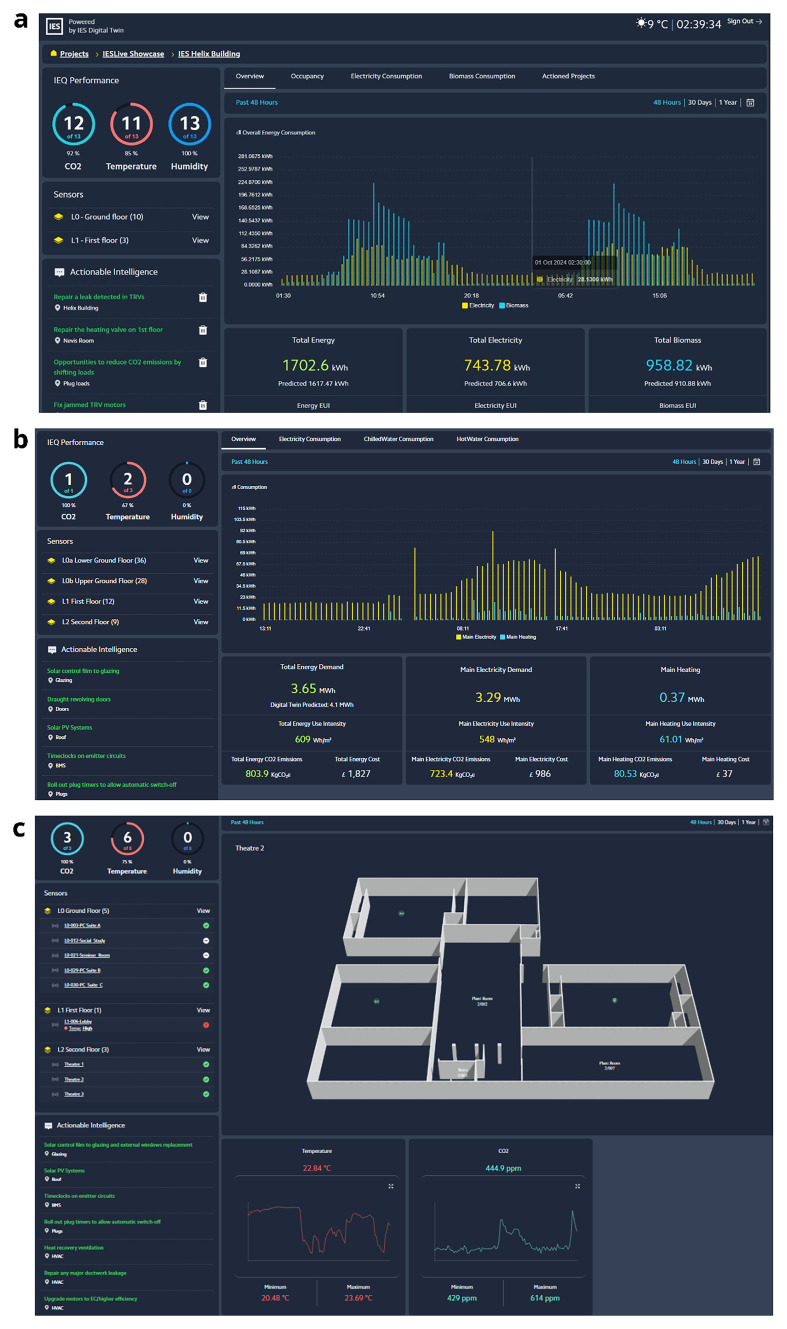
Digital twin dashboards displaying KPIs, live and historical data, and actionable intelligence within IES Live platform.

Finally, for the operational management tasks ( *Workflow 3 - optimized operation at building and building portfolio scales*) and the *Decarbonisation Roadmap* (step 5), the results are displayed using the IES Live on-the-cloud platform. As an iCIM platform, the IES Live platform is easily accessible through a URL, displaying real-time series data from IoT sensors crossed with the analysis of VE building-level simulation data. This type of display enables a PED manager to cope with operational tasks such as operational energy optimization, baseline and performance gap simulation, tracking and verifying actioned improvements, net-zero investment planning, investigating energy and carbon retrofitting scenarios, predicting future building performance, or simply providing a handy display of metered data and monitoring activities ( IES, 2024). Furthermore, as already mentioned in Figure 8, this tool can provide an overview of decarbonization roadmapping activities, further explained in Step 5 of this methodology. Three screenshots of the different IES Live dashboard tabs are included in Figure 11.

## Conclusions

This study fills a long-standing gap in urban energy planning by showcasing the potential of digital twinning technology to connect PED design and operation phases. By combining physics-based modeling, real-time data analysis, and user-centered design, the technique created under the TIPS4PED project advances a system-level approach. This all-encompassing viewpoint is crucial for the efficient design and administration of energy-positive urban areas, and makes a significant addition to the current European conversation on climate-neutral cities.

This study highlights the importance of coordinating technological advancements with the institutional and social aspects of PED deployment. A digital twin is a multidimensional interface for interaction and communication that goes beyond its function as a simple technical device. The platform's ability to provide clear, dynamic representations of complex situations encourages inclusive decision-making procedures and fosters openness and confidence among the participants. The core strategy of TIPS4PED's mission, which places high priority on human-centric principles, is the integration of simulation with stakeholder interaction.

In addition, the study highlights crucial scalability and interoperability. The structural complexity of installing PEDs in various metropolitan environments is reflected in the difficulties addressed, ranging from platform modularity to data heterogeneity. A major step towards developing a digital infrastructure that is reliable and flexible in a variety of situations has been taken with the efforts put into defining communication protocols, semantic models, and tool chain orchestration.

The proposed methodology provides a structured and integrated framework that addresses the multiscalar complexity of PEDs and offers tools for both strategic planning and real-time operational management. The case study of the Turin district demonstrates the practical feasibility of the approach, showing how digital twins can facilitate the integration of diverse energy systems and data sources, ultimately contributing to the achievement of a net positive energy balance.

One of the main contributions of this research is the development of a holistic digital twinning methodology that overcomes the limitations of conventional tools, typically focusing on building scale and relying on static data. By enabling real-time data integration, continuous monitoring, and predictive control, the proposed framework enhances the adaptability and responsiveness of urban energy systems, which is essential for the dynamic nature of PEDs. Furthermore, this approach emphasizes the central role of stakeholder engagement. Traditional PED planning processes often suffer from lack of coordination and limited inclusivity. The use of digital twins as interactive and visual platforms fosters greater transparency, improves communication across disciplines, and encourages the active participation of all relevant actors. This user-centric perspective is the key to increasing acceptance, aligning interests, and ensuring long-term project success. In addition to methodological advancements, this study identifies key technical and organizational barriers to the adoption of digital twinning in PED contexts. These include the need for interoperable infrastructure, cross-sectoral collaboration, and clear governance models. By addressing these issues, this study provides actionable insights for practitioners and policymakers who aim to implement digital twinning solutions in urban energy planning.

These results mark an important turning point in the TIPS4PED project research agenda, and will guide the adaptation of replication tools in other cities. According to this work, a new generation of PED support systems are both technically advanced and conceptually revolutionary. Digital twins could become key facilitators of Europe's sustainable urban transitions by reinventing methods used for city planning, simulation, monitoring, and engagement. The foundations laid here will be crucial in providing practical, scalable solutions for cities aiming to become climate-neutral by 2030, as the project moves into its next phase.

In conclusion, the results bridge a critical gap in existing PED support tools and contribute to advancing the state-of-the-art in urban energy management. The integration of digital twins into PED design and operation represents a promising pathway to enhance energy efficiency, sustainability, and stakeholder engagement, and future research will further delve into this direction and the specific steps of this methodology. Overall, as cities accelerate their transition towards carbon neutrality, this study offers a scalable and replicable model for future sustainable urban transformations.

## Ethics and consent

Ethical approval and consent were not required.

## Data Availability

This method article is based on project data collected within the Tips4PED project, involving local data gathered from multiple stakeholders and sources in Turin, and structured within IES software files. These data must remain private under each of the local partners’ consent. In the event that the specific data from the PED of Torino are requested by the reviewers or readers of this research, access must be granted under the explicit approval of the City of Torino and the Politecnico di Torino upon an express request to the corresponding author of this research via koldo.azcona@iesve.com.
