# Supplementary material for: Digital Twins for Positive Energy Districts: a 10-Step Method for Integrated Design and Optimized Operation
Source: Open Res Eur. 2025 Sep 23;5:294. [Version 1] doi: 10.12688/openreseurope.20962.1 (PMC12754371; doi:10.12688/openreseurope.20962.1)
Supplement: Peer review history

# Reviewer report: Reviewer response for version 1

**DOI:** 10.21956/openreseurope.22679.r61212

**Published:** 2025-12-30

**Authors:** Mari Hukkalainen

**Affiliations:** 1 VTT Technical Research Centre of Finland, Espoo, Finland

The article introduces a holistic digital twinning methodology that overcomes the limitations of conventional tools to supporting both the PED planning and operation. While it is good in overall and provides new insights to PED digital twin research, there are some minor issues that could improve the article. 

 It would be very interesting to understand what are the time intervals for data sets and energy performance scenario simulations used in this methodology (is e.g. energy demand data at daily/hourly/15-minutes time interval). Could you please add this information? Method section beginning states that annual data is used, but surely you also include shorter time steps in the energy optimisation steps? 

 Figure 1: the figure is not fully explained, e.g. what is the meaning of yellow borders at some of the boxes? Some of the texts are difficult to read as there is black text in the strongly coloured background. 

 Stakeholder roles are described in page 6, yet later the article mentions "PED manager", but such a role is not introduced/defined: who is this manager? a municipal expert? energy facility manager? I assume it depends case-by-case, but perhaps it would be helpful to explain what kind of role you see for the PED manager and who would it be. 

 Some of the references used are vague. E.g. at the beginning of introduction, authors claim that the main conceptual goal of a PED is widely accepted and use Gollner as reference, but the reference link guides to the JPI urban europe main page, and the real reference is not easily possible to find. There has been also discussion on the PED scientific community about whether the PED goal should focus on positive energy balance, and e.g. currently the DUT programme does not require that PEDs produce more renewable energy than they consume. Also Annex83 PED definition work could be referred here. 

 A minor issue is that the article claims very directly the importance of PEDs for climate neutral cities, but does not explain this relation well, and does not provide solid references or justification for this relation. E.g. the abstract claims that PEDs "are central to the European Union's vision for climate-neutral cities"; and similarly is stated in the last sentence of the Conclusions section ("Overall, as cities accelerate their transition towards carbon neutrality, this study offers a scalable and replicable model for future sustainable urban transformations", but in truth sustainable urban transformations is a much more broad topic than PEDs). Yes, PED concepts do contribute to this target, but the bold statement like this would need a justification or a reference; or making these claims more specifically focusing on district level sustainable energy system transition. There are quite a number of other energy system transition topics needed, in addition to PEDs, to fully decarbonise cities' energy systems. I would advise revising these sentences. 

 Minor question: before figure 7, it says "For example, the replacement of high-temperature heating distribution systems should occur before the installation of low-temperature heating generation systems (i.e., heat pumps)." Could you please explain why this should occur? 

 Figures 9 and 11: text is really small, hard to read at times. What does the letters a/b/c refer to in these figures? 

 pls check spelling: 3D-inmersive in Results section; CO2

 It is understandable that the detailed data is not shared from Turin case, this is not a problem as the article focuses on the methodology and technical architecture and process for the digital twin PED tool.

Is the rationale for developing the new method (or application) clearly explained?

Yes

Is the description of the method technically sound?

Yes

Are the conclusions about the method and its performance adequately supported by the findings presented in the article?

Yes

If any results are presented, are all the source data underlying the results available to ensure full reproducibility?

No

Are sufficient details provided to allow replication of the method development and its use by others?

Partly

Reviewer Expertise:

PED concept, demonstrations in real life, PED data and impact assessment; climate neutral cities and district level integrated energy planning.

I confirm that I have read this submission and believe that I have an appropriate level of expertise to confirm that it is of an acceptable scientific standard.

# Reviewer report: Reviewer response for version 1

**DOI:** 10.21956/openreseurope.22679.r64957

**Published:** 2025-12-16

**Authors:** Elena Malakhatka

**Affiliations:** 1 Chalmers University of Technology, Gothenburg, Sweden

1. Clearly explain how the proposed framework advances existing digital twin methodologies specifically within the Positive Energy District (PED) domain.

 2. Address how the digital twin informs or enables optimal control and support predictive control and operational optimization.

 3. The optimization algorithm is mentioned in the methodology steps, such as optimizing the demand profile based on predefined constraints (like minimizing energy costs) and adjusting simulated demand profiles to satisfy user requirements , and later in the optimized operational service section where an algorithm adjusts demand profiles based on user-defined constraints (e.g., maximizing self-consumption of renewables or minimizing costs). The methodological pipeline stops earlier, so these sections need to be more strongly demonstrated or integrated as a final, crucial step to address the limitation.

 4. The current methodology mentions UXR, including interviews, questionnaires, and a workshop, which is a good foundation. However, the framework does not sufficiently reflect the iterative and multi-stakeholder nature of PED development.

 5. The generative research focused primarily on energy managers of a university campus and municipal buildings, which are strong archetypes. To align better with the "long-term success" and "community benefits" of a PED, the UXR should be critically assessed and expanded to include:

**- Citizens/End-Users: **These are the residents in the residential assets and end-users of municipal services within the PED boundary. Their experience and input are essential for the long-term success of PEDs and for ensuring adoption.

**- Policymakers/Planners:** Their UXR is crucial to ensure the digital twin's reporting and visualization tools (iCIM/IES Live) meet their needs for strategic planning and decision-making beyond just energy management.

Is the rationale for developing the new method (or application) clearly explained?

Yes

Is the description of the method technically sound?

Partly

Are the conclusions about the method and its performance adequately supported by the findings presented in the article?

Yes

If any results are presented, are all the source data underlying the results available to ensure full reproducibility?

Partly

Are sufficient details provided to allow replication of the method development and its use by others?

Partly

Reviewer Expertise:

PED and UXR

I confirm that I have read this submission and believe that I have an appropriate level of expertise to confirm that it is of an acceptable scientific standard, however I have significant reservations, as outlined above.

# Reviewer report: Reviewer response for version 1

**DOI:** 10.21956/openreseurope.22679.r64961

**Published:** 2025-12-12

**Authors:** Xingxing ZHANG

**Affiliations:** 1 Dalarna University, Falun, Dalarna County, Sweden

This manuscript presents a case study by applying a digital twin framework for PED development. The methodological sequence is coherent, and the presented results are meaningful. There are few comments as below for the authors to consider:

- The manuscript has limited contribution in terms of innovation. The framework largely follows established practices for integrating real-time monitoring with simulation, and the manuscript does not clearly articulate how the approach advances existing digital twin methodologies within the PED domain. The authors are encouraged to clarify the novel components of their framework and specify what new insights or capabilities it enables beyond conventional tools.
- Another concern is the absence of a control or optimization component. Digital twins are typically characterized not only by data and simulation integration but also by their capacity to support predictive control, scenario evaluation, and operational optimization. In the current work, the methodological pipeline stops at the comparison of measured and simulated results, leaving the crucial final step underdeveloped, e.g., how the digital twin informs or enables optimal control. Addressing this limitation would significantly strengthen the practical relevance of the study.
- Furthermore, the framework does not sufficiently reflect the iterative and multi-stakeholder nature of PED development. PED planning processes rely heavily on co-creation, stakeholder dialogue, and iterative refinement. Incorporating outputs from workshops, consultations, or participatory design activities would enhance the completeness of the framework and better align it with contemporary PED methodologies. Such inputs could, for example, inform scenario definitions, behavioral assumptions, KPI selection, or boundary conditions within the digital twin.

Overall, the manuscript provides a useful technical overview. With these comments, the manuscript would benefit from (1) clearer articulation of methodological novelty, (2) inclusion or discussion of a control/optimization layer, and (3) better integration of stakeholder-driven, iterative design processes. These additions would substantially improve the contribution and robustness of the proposed framework.

Is the rationale for developing the new method (or application) clearly explained?

Yes

Is the description of the method technically sound?

Yes

Are the conclusions about the method and its performance adequately supported by the findings presented in the article?

Yes

If any results are presented, are all the source data underlying the results available to ensure full reproducibility?

Partly

Are sufficient details provided to allow replication of the method development and its use by others?

Yes

Reviewer Expertise:

Digitalisation of urban energy system, PED

I confirm that I have read this submission and believe that I have an appropriate level of expertise to confirm that it is of an acceptable scientific standard, however I have significant reservations, as outlined above.
